# Supplementary material for: Splice-Junction-Based Mapping of Alternative Isoforms in the Human Proteome
Source: Cell Rep. Author manuscript; Available in PMC 2020 Jan 15. (PMC6961840; doi:10.1016/j.celrep.2019.11.026)
Supplement: 2 [file NIHMS1546469-supplement-2.zip › DF1/PXD006675/CardiacFibroblast/CardiacFibroblast_10_PSMD2_GFGGSGSQVDSAR.pdf]

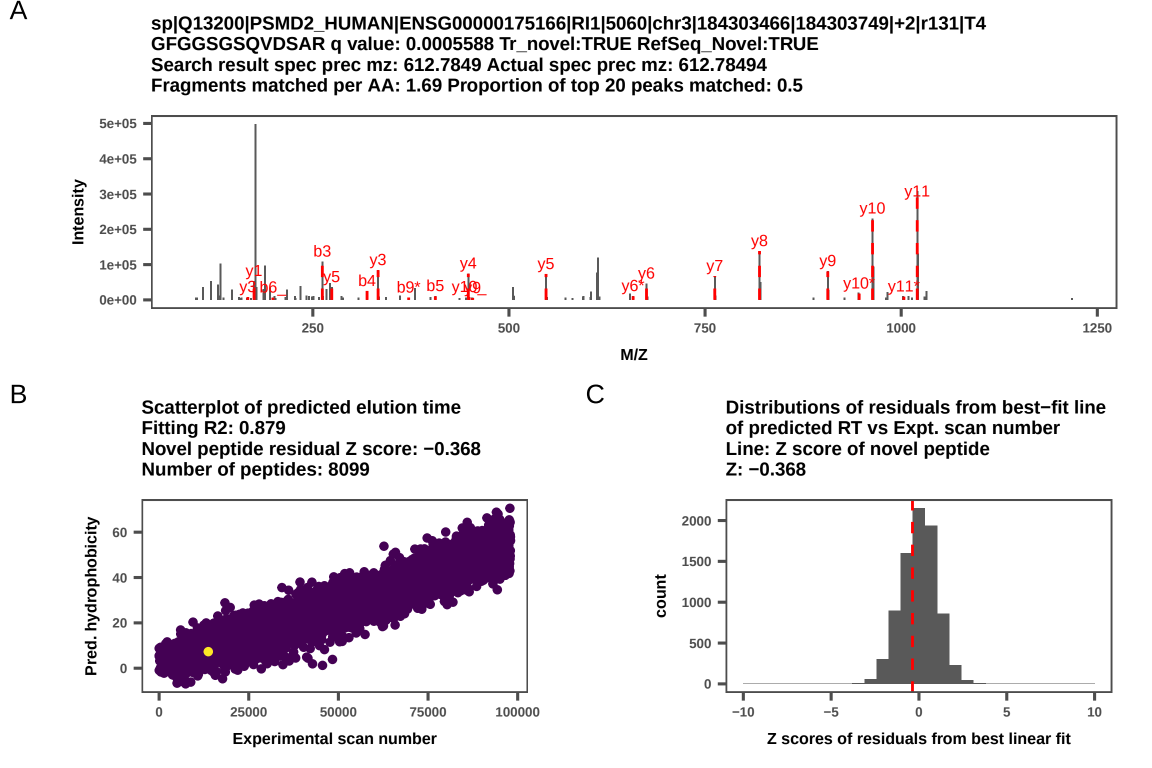
A
sp|Q13200|PSMD2_HUMAN|ENSG00000175166|RI1|5060|chr3|184303466|184303749|+2|r131|T4
GFGGSGSQVDSAR q value: 0.0005588 Tr_novel:TRUE RefSeq_Novel:TRUE
Search result spec prec mz: 612.7849 Actual spec prec mz: 612.78494
Fragments matched per AA: 1.69 Proportion of top 20 peaks matched: 0.5
Intensity
5e+05
4e+05
3e+05
2e+05
1e+05
0e+00
y1
y3
b6
b3
y5
y3
b4
b9*
b5
y4
y5
y6*
y6
y7
y8
y9
y10
y10*
y11
y11*
250
500
750
1000
1250
M/Z
B
Scatterplot of predicted elution time
Fitting R2: 0.879
Novel peptide residual Z score: −0.368
Number of peptides: 8099
Pred. hydrophobicity
60
40
20
0
0
25000
50000
75000
100000
Experimental scan number
C
Distributions of residuals from best−fit line
of predicted RT vs Expt. scan number
Line: Z score of novel peptide
Z: −0.368
count
2000
1500
1000
500
0
−10
−5
0
5
10
Z scores of residuals from best linear fit
